# Extraction and Characterization of Fiber and Cellulose from Ethiopian Linseed Straw: Determination of Retting Period and Optimization of Multi-Step Alkaline Peroxide Process

**DOI:** 10.3390/polym15020469

**Published:** 2023-01-16

**Authors:** Kibrom Feleke, Ganesh Thothadri, Habtamu Beri Tufa, Ali A. Rajhi, Gulam Mohammed Sayeed Ahmed

**Affiliations:** 1 Department of Manufacturing Engineering, School of Mechanical, Chemical and Materials Engineering, Adama Science and Technology University, Adama P.O. Box 1888, Ethiopia; 2 Department of Materials Engineering, School of Mechanical, Chemical and Materials Engineering, Adama Science and Technology University, Adama P.O. Box 1888, Ethiopia; 3 Department of Mechanical Engineering, College of Engineering, King Khalid University, Abha 61421, Saudi Arabia; 4 Centre of Excellence (COE) for Advanced Manufacturing Engineering, Program of Mechanical Design and Manufacturing Engineering, School of Mechanical, Chemical and Materials Engineering, ASTU, Adama P.O. Box 1888, Ethiopia

**Keywords:** linseed straw, fiber, cellulose, retting, extraction, optimization

## Abstract

Flax is a commercial crop grown in many parts of the world both for its seeds and for its fibers. The seed-based flax variety (linseed) is considered less for its fiber after the seed is extracted. In this study, linseed straw was utilized and processed to extract fiber and cellulose through optimization of retting time and a multi-step alkaline peroxide extraction process using the Taguchi design of experiment (DOE). Effects of retting duration on fiber properties as well as effects of solvent concentration, reaction temperature, and time on removal of non-cellulosic fiber components were studied using the gravimetric technique, Fourier transform infrared (FTIR) spectroscopy and thermal studies. Based on these findings, retting for 216 h at room temperature should offer adequate retting efficiency and fiber characteristics; 70% cellulose yield was extracted successfully from linseed straw fiber using 75% ethanol–toluene at 98 °C for 4 h, 6% NaOH at 75 °C for 30 min, and 6% H_2_O_2_ at 90 °C for 120 min.

## 1. Introduction

Agricultural crop by-products are considered inexpensive, abundant, annually renewable, and sustainable sources of fiber and cellulose. Finding alternate sources for present natural and synthetic fibers is critical for ensuring sufficient supply of fibers at reasonable rates in the future. Limitations in availability of land, water, and other resources required to grow natural fibers such as cotton, bamboo, sisal, hemp, and kenaf could limit availability and/or raise prices of those fibers, rendering them unaffordable for commodity applications. As a result, attempts to identify alternate fiber sources, particularly from cheap, copious, and renewable lignocellulose wastes, are considered extremely useful [1].

Flax (*Linum usitatissimum*) is a fibrous plant that is used as a commercial crop in milder climate regions [2]. Flax is produced for both its seeds and its fiber, although it is mostly farmed for its seeds, leading to its diversification into oilseed (linseed) and fibrous plant types [3]. The phenotypes and physiology of both types differ significantly from each other. Linseed may reach a height of 40–60 cm and has a highly branched stem, whereas the fibrous plant can reach a height of 80–120 cm and has a less branched stem [4]. Linseed and fibrous flax generate different amounts of stalks (30 and 85 dt ha^−1^, respectively), seeds (20 and 5 dt ha^−1^, respectively), and fiber (15 and 30%, respectively) [5]. In Ethiopia, flax is cultivated only for its seeds, which are used for oil, and the production capacity is on average 100,000 tons/year from around a 200,000 ha This ranked Ethiopia seventh among the top linseed producer countries in the world [6]. Many studies focus on flax fibers that are grown for textile applications and used to strengthen polymeric matrices [7]. However, fiber obtained from linseed straw every year has not been studied or promoted widely, even though properties of individual oilseed flax fibers [7,8,9], fiber extraction methods [10,11,12], and fiber and seed yields [5] are described comparatively in some studies. Subsequently, after seed harvest, linseed straw left in open fields is abandoned and/or burned, causing significant environmental harm. Many nations are now dealing with the problem of utilizing linseed straw [12].

Appropriate natural fiber extraction represents a major test faced during processing of plant fibers. Extraction methods to separate plant fibers include retting and mechanical extraction processes [13,14]. Common retting methods are water, dew, chemical, and enzyme retting [15,16]. Water retting is the most common method to extract high-quality fibers. In the retting process, existence of bacteria and moisture in the plants allows them to break down large parts from cellular tissues and adhesive substances that surround fibers, enabling separation of individual fibers from the plant. Depending on the fiber category, this process requires approximately 7 to 14 days [17,18]. Water retting is critical to processing of fibers; it influences qualities of generated fibers, and retting quality is a primary issue for industries that use natural fibers in their products [17]. Therefore, reaction time must be carefully evaluated when water retting is used, because under- or excessive retting can cause difficulties in separation of individual fibers, or may weaken fiber strength [19,20].

Cellulose is the most prevalent substance on the planet and the major component of plant-cell walls. The principal components of plant fibers include cellulose, hemicellulose, lignin, pectin, and wax [21,22]. However, cellulose is dependent on plant type and the geographical area in which it is cultivated [23]. As a result, suggests development of novel cellulose-based materials and demands detailed examination of their physical and chemical properties [24]. In this context, there has been a surge in interest in extracting cellulose from natural fibers in recent years. Several experiments have been conducted, utilizing natural fibers, including sisal, rice husk, sugarcane bagasse, cotton, hemp, jute, bamboo, and kenaf, among others, as sources of cellulose [23,25,26,27]. 

Extraction processes and methods for extracting cellulose vary and may include acid and alkaline media; each process yields unique properties for each type of cellulose produced. Because amount of cellulose and extraction process vary from plant to plant, it is crucial to note that cellulose tests and investigations are carried out separately [23]. The purpose of the extraction process is to obtain cellulose with a high yield and purity through elimination of non-cellulosic components—mainly extractives, hemicellulose, and lignin—using different solvents with various concentrations, reaction temperatures, and times for each step of extraction. This allows for extraction-process variable adjustments that lead to optimization strategies [23]. Therefore, in this study, the retting-time duration endpoint and influence of retting-time duration on fibers’ physical, tensile, and thermal properties were investigated. Chemical compositions of fiber retted at optimal water-retting duration were analyzed, and cellulose was extracted and characterized using optimized multistep cellulose-extraction-process parameters.

## 2. Materials and Methods

### 2.1. Materials

Chemicals utilized were ethanol LR (96%; Wasse Pharma PLC, Addis Ababa, Ethiopia), toluene AR (99.9%; Blulux Laboratories Pvt. Ltd., Faridabad, India), sulfuric acid AR (98%; Loba Chemie Pvt. Ltd., Mumbai, India), sodium hydroxide pellets LR (98%; Blulux Laboratories Pvt. Ltd., India), and hydrogen peroxide solution LR (30%; Fine Chemical General Trading PLC, Addis Ababa, Ethiopia).

Processing and characterization equipment used were a Soxhlet apparatus (M500-mL flask, Soxhlet tube, 300 mm condenser, medium-porosity cellulose thimbles, heating mantle, chiller, pH meter (AD8000 pH/mV/EC/TDS/Temperature); Adwa Instruments, Inc., Szeged, Hungary), an electronic balance (JF-2004; Tsingtao Unicom-Optics Instruments Co., Ltd., Laixi, China), a Clifton drying oven (NE9-56S; Nickel-Electro Ltd., Weston-Super-Mare, UK), a box-type resistance furnace (SX-2.5-12; Taisite Lab science INC., New York, NY, USA), a shaking water bath (GFL 1083; GFL Water Baths, Hamburg, Germany), a vertical autoclave (AVI-017B; Avishkar International Pvt. Ltd., Mumbai, India), a single-fiber electronic strength tester (Fiber Tenso-Lab-331A; Mesdan-Lab, Raffa, Italy), a FTIR-6600 spectrometer (JASCO Inc. Ltd., Easton, MD, USA), and a thermogravimetric and differential thermal analyzer (TG-DTA, HCT-3; Beijing Henven Instruments, Beijing, China).

### 2.2. Fiber Extraction and Characterization Methods

A 50 g linseed straw bundle with a 20 cm length and a 3 mm mean stalk diameter was cut from the center section of the stems for uniformity of samples. Next, the bundled stalks were immersed in transparent plastic bottles, each filled with the same amount of water, with a 1:33 solid-to-liquid ratio [28]. After immersion, the bottles were stored at room temperature for 48–264 h, without lids, for retting, as shown in Table 1 [29,30].

About 275 mL of retted water was taken from all samples and tested every 24 h using a calibrated pH meter to find pH measurement [31,32]. Increase in weight of wetted linseed stalks (*m*_w_) from initial dry linseed stalk weight (*m*_d_) due to water absorption from each retting time was recorded, and stalk water-absorption percentage (*w*_a_%) was estimated based on Equation (1) [33,34].

(1) $$w_{a}\%=\left(\frac{m_{w}-m_{d}}{m_{d}}\right)\times 100$$

The percentage of mass change between dry non-retted (*w*_1_) and dry retted (*w*_2_) linseed stalks was used to calculate stalk weight loss (*w*_l_%) using Equation (2) [29,35].

(2) $$w_{l}\%=\left(\frac{w_{1}-w_{2}}{w_{1}}\right)\times 100$$

Weight of linseed fiber extracted (*m*_o_) from the initial linseed stalk weight (*m*_i_) was recorded for each retting time, and the fiber yield percentage (*Y*%) was determined using Equation (3) [28,36].

(3) $$Y\%=\left(\frac{m_{o}}{m_{i}}\right)\times 100$$

Density of oven-dried stalks amounted to a constant weight [34,37,38]; non-, under-, optimally, and over-retted linseed fiber bundles were measured for distilled water density (*ρ*_w_) using the liquid pycnometer technique. Mass of an empty pycnometer (*m*_1_), a pycnometer filled with chopped fibers (*m*_2_), a pycnometer filled with water (*m*_3_), and a pycnometer filled with water and chopped fibers (*m*_4_) was measured and calculated. Density of fibers (*ρ*_f_) was estimated based on Equation (4) [38,39].

(4) $$\rho_{f}=\left(\frac{m_{2}-m_{1}}{\left(m_{3}-m_{1}\right)-\left(m_{4}-m_{2}\right)}\right)\times \rho_{w}$$

Diameter of linseed fibers was measured using an optical microscope. Three samples, from under-, optimally, and over-retted fibers, were prepared; then three equidistant points were marked on a single fiber, and the diameters of these points were measured with two replications. The average diameter was then calculated; this could be considered the mean diameter of the fibers [40].

Fiber samples with different degrees of water retting were kept in the open air at room temperature and 55–65% RH for one week. Then, after drying in an oven to a constant weight, weights of wetted (*W*_w_) and oven-dried (*W*_d_) samples were measured. Moisture content percentage (*M*_C_%) of the fiber was calculated using Equation (5) [33,34].

(5) $$M_{C\;}\%=\left(\frac{W_{w}-W_{d}}{W_{w}}\right)\times 100$$

A tensile test of single fibers with different retting durations was performed according to ASTM D 3822–07, using a single-fiber electronic strength tester. The test was carried out at room temperature and 65% RH, with a gauge length of 100 mm, a load range of 5 N, and a test speed of 100 mm/min [41]. 

**Figure 1:**
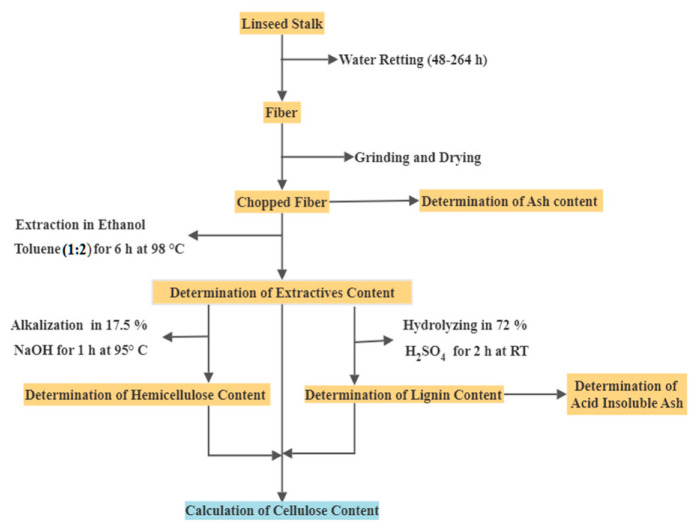
Flowchart of retting and chemical-composition analysis.

A chemical composition analysis (dry-weight basis) of the fiber was conducted to quantify mainly the percentage amounts of cellulose, hemicellulose, lignin, and extractives. This analysis used 1:2 ethanol–toluene for 6 h at 98 °C in a Soxhlet apparatus to determine extractive content [42,43,44,45], 17.5% NaOH at 95 °C for 60 min in a reciprocating water bath to quantify hemicellulose content [46,47], and 72% H_2_SO_4_ at room temperature for 120 min; hydrolyzed samples were diluted with distilled water to a 3% acid concentration (adapted from TAPPI T-222 om-02) [48]. Samples were autoclaved for 1 h at 121 °C and cooled for about 20 min at room temperature; the diluted suspensions were centrifuged at 5000 rpm for 15 min and vacuum-filtered. The residues were burned in a muffle furnace at 550 ℃ for 3 h to quantify the amount of ash in the acid-insoluble lignin [41,49], as shown in Figure 1.

Percentage amounts of non-cellulosic constituents (*wt*.%) were calculated from the difference between initial (*w*_i_) and final (*w*_f)_ fiber weights, using the gravimetric method based on Equation (6) [50,51].

(6) $$wt.\%=\left(\frac{w_{i}-w_{f}}{w_{i}}\right)\times 100$$

The ash content of dry, chopped raw fiber was determined via burning in a 550 °C furnace for 4 h [48], allowance to cool to room temperature in a desiccator, and weighing (adapted from TAPPI (T 211 om-02)). Finally, the cellulose percentage in *wt.*% of biomass was calculated using Equation (7), assuming that extractives, hemicellulose, lignin, ash, and cellulose were the main chemical compositions of the linseed fiber [52,53,54].

(7) Cellulose%=100%−(Extractives+Hemicellulose+Lignin+Ash)%

### 2.3. Cellulose Extraction and Characterization Methods 

**Figure 2:**
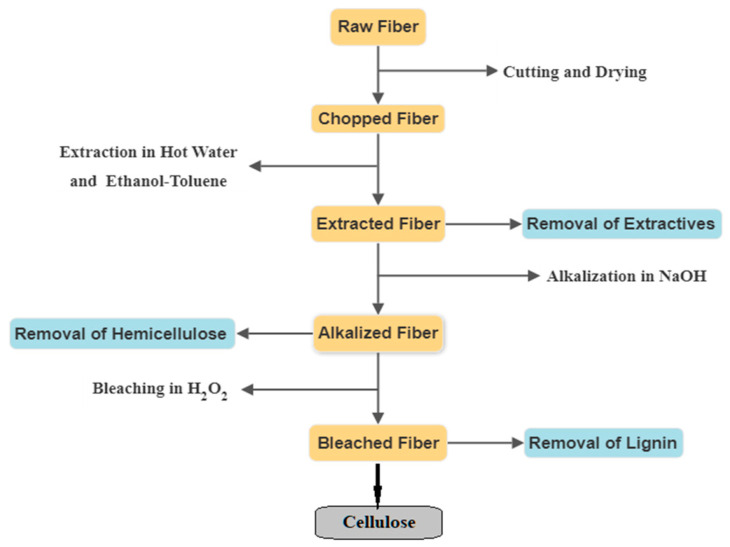
Flowchart of the cellulose-extraction process.

Cellulose was extracted from linseed straw through sequential chemical treatments to remove extractives, hemicellulose, and lignin, as shown in Figure 2.

Removal percentage of non-cellulosic components (*R*%) of linseed fiber in each step of the cellulose extraction process was calculated via taking the weight difference between initial (*m*_i_) and final (*m*_f_) fiber weights, using the gravimetric method based on Equation (8).

(8) $$R\%=\left(\frac{m_{i}-m_{f}}{m_{i}}\right)\times 100$$

### 2.4. Fourier Transform Infrared (FTIR)

In the FTIR analysis, FTIR spectra of the fibers (non-retted, retted, extracted, alkalized, and bleached) were recorded using a FT–IR spectrometer with a 2 mm/s scanning speed and a 4 cm^−1^ resolution in a range of 400–4000 cm^−1^ wavenumbers [48,55,56,57]. 

### 2.5. Thermogravimetric Analysis (TGA)

In the thermal study, thermal degradation characteristics of fibers (non-retted, retted, extracted, alkalized, and bleached) were analyzed using a thermogravimetric and differential thermal analyzer, from room temperature to 700 °C, at a heating rate of 20 °C min^−1^ in N_2_ atmospheres, and using an 8 mg sample weight [1,58,59,60].

### 2.6. Statistical Method for Optimization

The selected factors (concentration, temperature, and time) and levels (low, medium and high) for the extraction processes are shown in Table 2. The Taguchi *L*_9_ Orthogonal Array (OA) design of experiments was employed to investigate contribution of selected extraction conditions (concentration of solvents, reaction temperature, and time) to yield removal of extractives, hemicellulose, and lignin, as shown in Table S1. 

## 3. Results and Discussion

### 3.1. Retted Water pH, Stalk Water Absorption and Stalk Weight Loss Analysis

**Figure 3:**
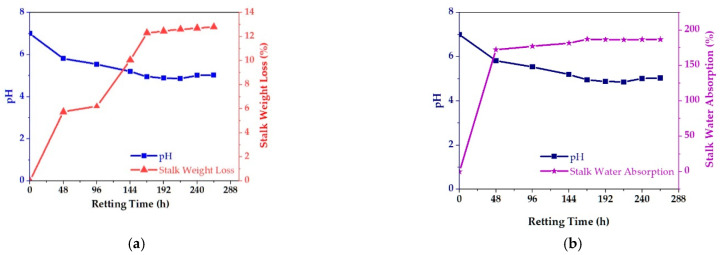
(**a**) Retting water pH with stalk weight loss. (**b**) Retting water pH with stalk water absorption at different retting times.

Figure 3a,b illustrate the effect of water-retting time on pH value of retting water, stalk water absorption, and stalk weight loss. When the pH value of the retted water dropped from 7.00 to 4.86, after 216 h, it remained steady and then increased again.

Many bacteria isolated from bast fibers are capable of promoting retting. The most important phase of this process is hydrolysis of pectic matter that surrounds and cements fibers, thereby loosening fibers from the stem and helping to extract those fibers [31]. Due to absence of pectic matter to be hydrolyzed and utilization of D-galacturonic acid (GA) by bacteria, concentration of GA, which is the end product of bacterial hydrolysis in retting water, began to fall [31,32]. 

The effect of retting-time duration on the water absorption percentage of linseed stalks showed that water absorption increased rapidly to 172.47% during the first 48 h, then increased slowly to the equilibrium water-absorption percentage of 187.21% at 168 h. After this immersion-time duration had passed, percentage of water absorption became stable, which means less than 1% of variations were observed, and no more weight gain of the wetted linseed stalk was observed during retting-time increments, as shown in Figure 3b. Forty-eight hours after equilibrium water absorption was the optimum retting time for successful fiber extraction.

Water retting occurs when water penetrates the center-stalk section of the plant, swelling the interior cells and shattering the outermost layer to enhance absorption of water and produce bacteria that promotes retting [61,62]. Therefore, retting up to equilibrium water absorption helps with removal of pectin and successful fiber extraction during water retting. 

Extending duration of water retting resulted in a considerable increase in weight loss, as can be shown in Figure 3a. When the duration was extended from 48 to 168 h, the weight loss increased from 5.74 to 12.3% due to removal of impurities and pectin components of the fiber. From 168 to 216 h, weight-loss variations were nearly stable or less than 1%, and at the end of this time, 12.67% fiber yield was obtained. However, after 216 h of retting, weight loss was slightly increased due to removal of other non-cellulosic constituents [35,63,64]. As a result, the retted water pH, stalk water absorption, and stalk weight loss values obtained can be used to predict optimal retting time.

### 3.2. Effect of Retting Duration on Fiber Properties

**Figure 4:**
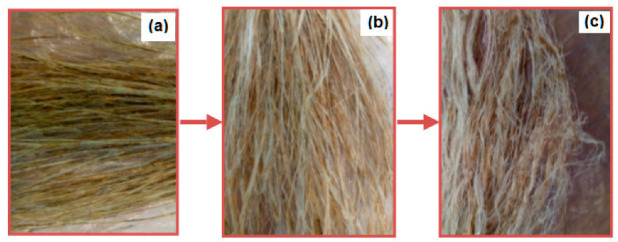
Images of (**a**) under-, (**b**) optimally, and (**c**) over-retted fibers.

Effects of retting duration on physical and tensile properties of fibers that were extracted under different retting durations, shown in Figure 4, were investigated.

#### 3.2.1. Physical Properties 

Table 3 demonstrates effects of retting duration on physical properties—mainly diameter, density, and moisture—of (R_0_) non-retted, (R_1_) under-retted, (R_2_) optimally retted, and (R_3_) over-retted fibers.

These results revealed that as degree of retting increased, average diameter of the fiber reduced due to removal of surface components via retting [65,66]. Initially, an increment in density values was observed, with increasing retting degree due to removal of less-dense constituents and impurities such as pectin [67,68], but over-retted fibers showed a relative reduction in density as a result of cell-wall decompression [69,70]. 

The mean density of optimal retted fiber was 1.52 g/cm^3^ and the density values of flax fiber reported in previous works of the literature were from 1.40 to 1.55 g/cm^3^ [71,72]. These values were obtained with different methods, such as a helium pycnometer (1.54 g/cm^3^) [73], a gas pycnometer (1.49 to 1.52 g/cm^3^) [74], and immersion in water (1.54 g/cm^3^) [75]. Moisture content was reduced with increasing retting degree due to the high amount of cortical parenchyma components remaining on the surface of non-retted and under-retted fibers; these fibers may have high water interaction [76]. 

#### 3.2.2. Tensile Properties

The effect of retting duration on tensile properties—specifically breaking force; breaking elongation; and tenacity of (R_1_) under-retted, (R_2_) optimally retted, and (R_3_) over-retted fibers—were tested as shown in Table 3. Initially, mean breaking force and tenacity of each single fiber were enhanced due to removal of a larger amounts of weak substances, such as pectin and other impurities; results were reduced with further increments of retting duration due to cellulose-component degradation resulting in presence of more weak spots and reduction in diameter of the fibers [65,77,78]. 

These results showed that mean elongation values decreased with an increasing degree of retting due to removal of non-cellulosic components; this tends to result in fiber brittleness [30,79]. 

### 3.3. Chemical Composition Analysis

The primary chemical compositions of linseed straw are cellulose, hemicellulose, lignin, and extractives. The chemical constituents of linseed fiber are 68, 20, 5, 4, and 3% of cellulose, hemicellulose, lignin, extractives, and ash, respectively. Cellulose content is comparatively higher than are different lignocellulose biomasses from agricultural wastes, as shown in Table 4.

These variations may be due to the type of agricultural crop and the geographic area where the plants were cultivated; the amount of these constituents might vary even among the same plants. Uniquely, all fibers contain the same constituents, but in different percentages, which results in different behaviors [83].

### 3.4. Cellulose Extraction, Characterization and Optimization

**Figure 5:**
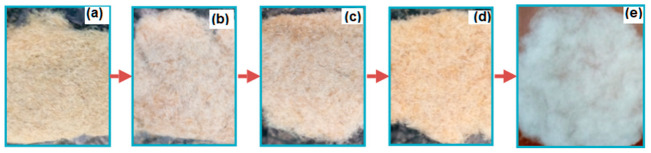
Images of (**a**) non-retted, (**b**) retted, (**c**) dewaxed, (**d**) alkalized, and (**e**) bleached fibers.

The cellulose extraction process was conducted in multi-step extraction processes via optimization of extraction-process conditions, including solvent concentration, reaction temperature, and time. The extraction steps mainly focused on removal of extractives, hemicellulose, and lignin using ethanol–toluene, sodium hydroxide, and hydrogen peroxide solvents, respectively, under different extraction-process conditions. The results for a fiber after each step of extraction are shown in Figure 5.

#### 3.4.1. Statistical Analysis 

The experimental results showed that the maximum removal values of extractives, hemicellulose, and lignin were 4.90, 18.10, and 4.00% respectively; these results were observed at extraction-variable combinations of 75% at 98 °C for 4 h, 6% at 75 °C for 30 min, and 6% at 90 °C for 120 min, respectively. However, the predicted mean removal (%) of extractives, hemicellulose, and lignin tested under optimum values from the signal to noise ratio (SNR) graph was calculated as 4.88, 18.25, and 4.19% at extraction-variable combinations of 75% at 98 °C for 8 h, 6% at 75 °C for 90 min, and 10% at 90 °C for 120 min, with error values of 0.40, 0.82, and 4.53%, respectively. This indicates that the experimental and predicted results were in good agreement, as shown in Table 5. 

The ANOVA results shown in Tables S2–S4 explain the significant level, contribution percentage, and rank of each factor in removal of extractives, hemicellulose, and lignin, respectively. The concentrations of the solvents in removal of extractives, hemicellulose, and lignin were statistically significant (*p* ≤ 0.05), contributing 97.25, 64.5, and 81.47% to the response, respectively. The reaction temperatures in removal of extractives and hemicellulose were statistically significant, contributing 2.74 and 35.09%, respectively. This value, however, was not significant in removal of lignin (*p* ≥ 0.05), contributing only 7.48%. The reaction time in removal of extractives, hemicellulose, and lignin was not significant, with no contribution, 0.29%, and 9.26%, respectively.

The ANOVA findings of the linear model equations shown in Equations (S1)–(S4) can appropriately explain removal of extractives, hemicellulose, and lignin within a wide range of operating circumstances, with coefficients of determination (R^2^) of 0.569, 0.879, and 0.8974 at a 95% level of confidence. The response models examined in this study can explain removal of extractives, hemicellulose and lignin; they contributed 56.95, 87.92, and 89.74% to the response, respectively. 

#### 3.4.2. Fourier Transform Infrared (FTIR) Analysis 

**Figure 6:**
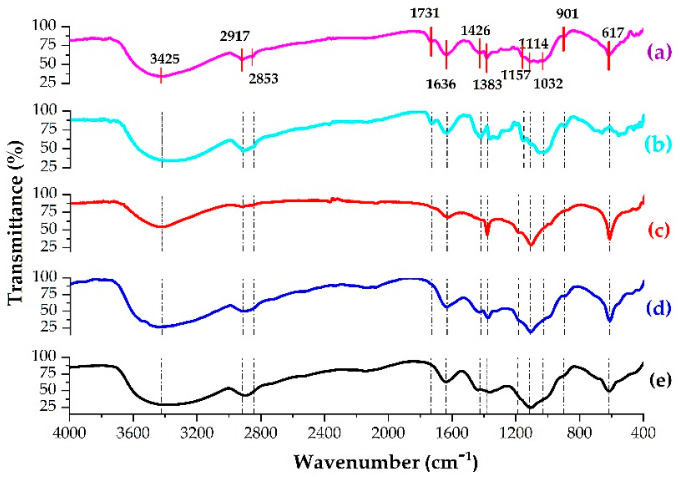
The FTIR spectra of (**a**) non-retted, (**b**) retted, (**c**) dewaxed, (**d**) alkalized, and (**e**) bleached fibers.

The FTIR spectra of the non-retted, retted, extracted, alkalized, and bleached linseed fibers shown in Figure 6 were interpreted and discussed according to reported studies regarding the sources of FTIR peaks and their assignments, as shown in Table 6. For every stage of the extraction procedure, FTIR analysis was carried out to identify presence of chemical functional group changes [84]. All samples presented two main absorbance regions: the fingerprint region (700–1800 cm^−1^) and the functional group region (2700–3500 cm^−1^). However, specific absorption peaks can be identified for each particular component [42]. 

The presence of nearly similar functional groups at 3,425, 2,917, 1,636, 1,114, and 617 cm^−1^ in all fibers justified preservation of the basic chemical structure of cellulose fiber and water, even after all treatments during the extraction process. It was expected that during the extraction process, non-cellulosic components of the fiber—hemicellulose, lignin, and extractive (pectin, wax) contents—could be completely or partially removed. Therefore, corresponding absorption peaks to these components disappeared or diminished in intensity value. The treatments, on the other hand, increased intensity of bands that corresponded to cellulose [85]. 

The absorption peaks that corresponded to the extractives were 2917, 2853, 1114, and 1032 cm^−1^. The absorbance peak at 2853 cm^−1^ disappeared, and others were reduced after retting and extraction due to removal or reduction of pectin and wax. The absorption peaks at 3425, 2917, 1732, 1426, 1383, 1114, 1032 and 901 cm^−1^ are related to hemicellulose. The absorption peak at 1731 cm^−1^ disappeared, and the others were diminished in peak intensity because of hemicellulose removal during the alkalization process. Absorption peaks observed at 3425, 2917, 1731 and 1426 cm^−1^ are associated with lignin. The absorption peak at 1731 cm^−1^ disappeared, and the others diminished in peak intensity because of lignin removal during the bleaching process.

#### 3.4.3. Thermogravimetric Analysis (TGA)

**Figure 7:**
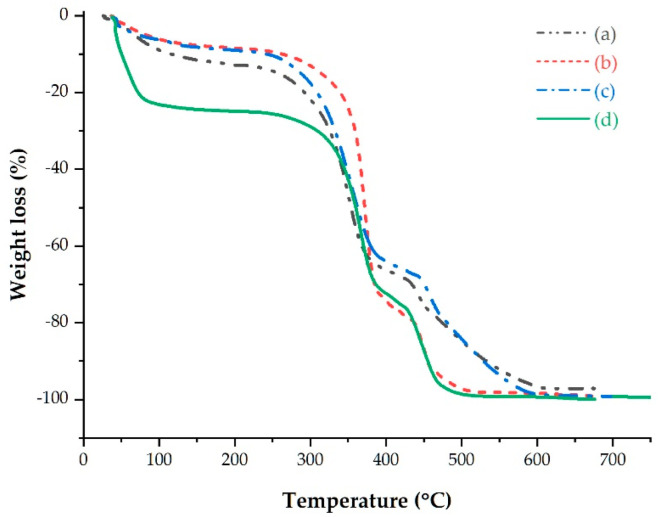
TGA graphs of (**a**) raw, (**b**) dewaxed, (**c**) alkalized, and (**d**) bleached fibers.

Decomposition of lignocellulose materials mainly shows three stages of degradation; due to differences in chemical structures between extractives, hemicellulose, cellulose, and lignin, they usually decompose at different temperatures [77,88]. Degradation of linseed straw fiber went through three phases, as shown in Figure 7: light-component drying and evaporation, hemicellulose and amorphous cellulose decomposition, and crystalline cellulose and lignin decomposition. 

Weight loss, decomposition temperature ranges, and residue contents of fibers in each degradation stage are summarized in Table 7.

The thermal stability of the raw, extracted, alkalized, and bleached fibers was 179, 205, 250, and 269 °C, respectively. All extraction steps resulted in thermal stability improvement of the fiber due to retention and improvement of the structural order, as well as reduction in amorphous content [89]. 

Each fiber showed dissimilar weight losses and temperature ranges in all degradation stages. Weight loss (%) in the first stage was higher for raw fiber compared to that of extracted and alkalized fibers due to presence of extractives and higher moisture content. It was reduced after removal of extractives and hemicellulose, which are responsible for moisture absorption of fibers, and on the other hand, the bleached fiber showed the highest weight loss (%) due to more moisture-absorption properties of the fiber after removal of lignin, which is naturally hydrophobic [90]. 

An increase in weight loss was observed in the second degradation stage after the extraction process due to the removal of extractives, which increased the proportions of cellulose and hemicellulose [77]. However, weight loss decreased after the alkalization and bleaching processes due to removal of hemicellulose and lignin. The decomposition temperature range decreased after each extraction step in all stages, since degradation of non-cellulosic components occurred over a low, broad temperature range due to presence of low molecular weight components [91]. Finally, residues that corresponded to ash content decreased during the extraction steps as a result of removal of non-cellulosic matter, which is responsible for ash content [92,93].

## 4. Conclusions

The linseed plant is a dual-purpose crop. Even if it is first and foremost cultivated for its seeds, its straw can be also useful, possibly contributing to an additional source of income for farmers as a source of fiber and cellulose due to its comparable bast-fiber and cellulose contents. This study reported optimum extraction of fiber and cellulose, as well as characterization from linseed straw. According to the experiments and analyses performed, pH, stalk water absorption, and weight loss were found to be good indicators for termination time of the water-retting process and optimum retting time. Effects of retting-time duration on tensile and physical properties of the fibers were tested, analyzed, and discussed. At the recommended optimum retting time (216 h), fibers with a density of 1.52 g/cm^3^, a diameter of 104.65 µm, and a moisture content of 8.32% had a mean breaking force of 278.4 cN, a breaking elongation of 2.06%, and a tenacity value of 59.1 cN/tex. The chemical composition of the optimum retted fiber had content of 68% cellulose, 20% hemicellulose, 5% lignin, 4% extractives, and 3% ash. Cellulose was present at the highest levels; therefore, extraction of cellulose from linseed straw is feasible and a promising sustainable cellulose source for different applications, such as packaging, filtration, composites, implants, paper, and pulp. Cellulose is extracted through successful optimization of multi-step extraction-process parameters for linseed straw. The recommended optimum cellulose extraction conditions for linseed fiber were identified as 75% ethanol–toluene at 98 °C for 4 h, 6% NaOH at 75 °C for 30 min, and 6% H_2_O_2_ at 90 °C for 120 min, for successful removal of non-cellulosic constituents.

## Figures and Tables

**Table 1:** Sample names and corresponding retting-time durations.

Sample	RT_0_	RT_1_	RT_2_	RT_3_	RT_4_	RT_5_	RT_6_	RT_7_	RT_8_
Retting Time (h)	0	48	96	144	168	192	216	240	264

**Table 2:** Factors and levels of solvents for extraction processes.

Solvent	Factors (Parameters)								
	Concentration (%)			Temp. (°C)			Time (h)		
	Level			Level			Level		
	−1	0	+1	−1	0	+1	−1	0	+1
C_2_H_5_OH:C_7_H_8_	50	75	100	78	88	98	4	6	8
NaOH	2	6	10	50	62.5	75	1/2	1	1 & 1/2
H_2_O_2_	2	6	10	80	90	100	1	1 & 1/2	2

−1, 0, and +1 are levels related to low, medium, and high values of the selected extraction process variables, respectively.

**Table 3:** Summary of the mean values of tensile and physical properties of fibers.

Sample	Tensile Properties			Physical Properties		
	Breaking Force (cN)	Breaking Elongation (%)	Tenacity (cN/tex)	Diameter (µm)	Density (g/cm^3^)	Moisture (%)
R_0_	-	-	-	-	1.33	9.34
R_1_	219.9	2.17	41.7	128.22	1.43	8.57
R_2_	278.4	2.06	59.1	104.65	1.52	8.32
R_3_	193.4	1.73	54.6	90.36	1.41	7.72

**Table 4:** Chemical composition of lignocellulose biomasses from agricultural wastes.

Lignocellulose Biomass	Cellulose (%)	Hemicellulose (%)	Lignin (%)	Extractives (%)	References
Linseed Straw	68	20	5	4	This study
Oleaginous Flax	47	24	21	-	[9]
Sugar Bagasse	43.6	33.5	18.1	3.1	[80]
Corn Cob	45	35	15	5	[81]
Corn Stover	40	25	17	18	[81]
Rice Straw	38.3	31.6	18.8	11.3	[82]

**Table 5:** Experimental and predicted removals of non-cellulosic components of fiber.

Run	Variables			Response (%)								
	C (%)	T (°C)	t (h)	Extractive Removal			Hemicellulose Removal			Lignin Removal		
				EXP	PRED	ERR	EXP	PRED	ERR	EXP	PRED	ERR
1	−1	−1	−1	2.52	2.40	0.12	10.0	9.81	0.19	1.6	1.45	0.15
2	−1	0	0	2.67	3.88	−1.21	11.9	13.72	−1.82	2.0	2.46	−0.46
3	−1	+1	+1	2.85	2.73	0.12	13.7	13.42	0.28	2.6	2.63	−0.03
4	0	−1	0	4.54	3.98	0.56	14.5	13.40	1.10	3.0	3.12	−0.12
5	0	0	+1	4.69	4.15	0.54	16.2	15.46	0.74	4.0	3.40	0.60
6	0	+1	−1	4.90	4.35	0.55	18.1	17.18	0.92	3.2	2.96	0.24
7	+1	−1	+1	3.50	3.86	−0.36	14.4	14.85	−0.45	3.6	3.71	−0.11
8	+1	0	−1	3.65	4.00	−0.35	15.3	15.84	−0.54	3.8	3.73	0.07
9	+1	+1	0	3.83	4.18	−0.35	17.8	18.16	−0.36	3.4	3.69	−0.29

**Table 6:** Sources of FTIR peaks and their assignments [1,84,86,87].

Wavenumber (cm^−1^)	Bond	Vibration	Sources
3425	O-H	Stretching	Cellulose, hemicellulose, lignin, and pectin
2917	C-H, C-H_2_	Stretching	Cellulose, hemicellulose, lignin, pectin, wax, and fat
2853	C-H_2_	Symmetric Stretching	Wax
1731	C=O	Unconjugated	Hemicellulose and lignin
1636	O-H	Stretching	Absorbed water
1426	O-H, C-H	Bending	Cellulose, hemicellulose, and lignin
1383	COO^-^	Stretching	Hemicellulose
1157	C-O-C	Asymmetric Stretching	Cellulose, hemicellulose, and lignin
1114	C-O	Stretching	Cellulose, hemicellulose, and pectin
1032	C-O-C	Bending	Cellulose, hemicellulose, pectin, wax, and fat
901	C-O-C	Stretching	Cellulose and hemicellulose
617	C-OH	Out-of-Plane Bending	Cellulose

**Table 7:** Thermogravimetric analysis of fibers at different extraction steps.

Sample	1st Stage		2nd Stage		3rd Stage		Ash (%)
	Wt. Loss (%)	T (°C)	Wt. Loss (%)	T (°C)	Wt. Loss (%)	T (°C)	
Raw Fiber	11.36	26–145	56.26	179–426	28.46	426–609	3.00
Extracted Fiber	7.51	37–139	70.45	205–438	18.79	430–510	2.25
Alkalized Fiber	7.44	38–121	57.20	250–440	30.69	440–591	1.72
Bleached Fiber	23.50	37–110	49.89	269–427	22.55	427–510	1.13

## Supplementary Materials

The following supporting information can be downloaded at: https://www.mdpi.com/article/10.3390/polym15020469/s1. Table S1: Taguchi L9 orthogonal array layout for cellulose extraction. Table S2: ANOVA results for removal of extractives (%) under different extraction conditions. Table S3: ANOVA for removal of hemicellulose (%) under different extraction conditions. Table S4: ANOVA for removal of lignin (%) under different extraction conditions.
