# Supplementary material for: Extraction and Characterization of Fiber and Cellulose from Ethiopian Linseed Straw: Determination of Retting Period and Optimization of Multi-Step Alkaline Peroxide Process
Source: Polymers (Basel). 2023 Jan 16;15(2):469. doi: 10.3390/polym15020469 (PMC9912263; doi:10.3390/polym15020469)
Supplement: Supplementary file 1 [file polymers-15-00469-s001.zip › polymers-2026647-SI.pdf]

polymers MDPI

Supplementary Information

Extraction and Characterization of Fiber and Cellulose from Ethiopian Linseed Straw: Determination of Retting Period and Optimization of Multi-Step Alkaline Peroxide Process

Kibrom Feleke [1], Ganesh Thothadri [2,*], Habtamu Beri Tufa [1], Ali A. Rajhi [3] and Gulam Mohammed Sayeed Ahmed [1,4]

[1] Department of Manufacturing Engineering, School of Mechanical, Chemical and Materials Engineering, Adama Science and Technology University, P.O. Box 1888, Adama, Ethiopia; kbmat17@gmail.com (K.F.); habtamu.beri@astu.edu.et (H.B.T.); drgmsa786@gmail.com (G.M.S.A.)
[2] Department of Materials Engineering, School of Mechanical, Chemical and Materials Engineering, Adama Science and Technology University, P.O. Box 1888, Adama, Ethiopia
[3] Department of Mechanical Engineering, College of Engineering, King Khalid University, Abha 61421, Saudi Arabia; arajhi@kku.edu.sa
[4] Centre of Excellence (COE) for Advanced Manufacturing Engineering, Program of Mechanical Design and Manufacturing Engineering, School of Mechanical, Chemical and Materials Engineering, ASTU, P.O. Box 1888, Adama, Ethiopia
* Correspondence: ganesh_reliez@yahoo.co.in

Table S1. Taguchi L_9 orthogonal array layout for cellulose extraction.

Experimental Run	Concentration (%)	Temperature (°C)	Time (h)
1	-1	-1	-1
2	-1	0	0
3	-1	+1	+1
4	0	-1	0
5	0	0	+1
6	0	+1	-1
7	+1	-1	+1
8	+1	0	-1
9	+1	+1	0

Table S2. ANOVA results for removal of extractives (%) under different extraction conditions.

Source	DF	Seq SS	Adj SS	Adj MS	F	P	Contribution (%)	Rank
Conc.	2	6.18380	6.18380	3.09190	30919.00	0.000	97.25	1
Temp.	2	0.17420	0.17420	0.08710	871.00	0.001	2.74	2
Time	2	0.00020	0.00020	0.00010	1.00	0.500	0.00	3
Residual Error	2	0.00020	0.00020	0.00010			0.00	
Total	8	6.35840					100	

Table S3. ANOVA for removal of hemicellulose (%) under different extraction conditions.

Source	DF	Seq SS	Adj SS	Adj MS	F	P	Contribution (%)	Rank
Conc.	2	35.2822	35.2822	17.6411	260.28	0.004	64.35	1
Temp.	2	19.2422	19.2422	9.6211	141.95	0.007	35.09	2
time	2	0.1622	0.1622	0.0811	1.20	0.455	0.29	3
Residual Error	2	0.1356	0.1356	0.0678				
Total	8	54.8222						

Table S4. ANOVA for removal of lignin (%) under different extraction conditions.

Source	DF	Seq SS	Adj SS	Adj MS	F	P	Contribution (%)	Rank
Conc.	2	4.14927	4.14927	2.07463	45.80	0.021	81.47	1
Temp.	2	0.38127	0.38127	0.19063	4.21	0.192	7.48	2
time	2	0.47167	0.47167	0.23583	5.21	0.161	9.26	3
Residual Error	2	0.09060	0.09060	0.04530				
Total	8	5.09280						

Based on the multiple linear regression model chosen to fit the data, the relations between the non-cellulosic components removal percentage (*R* %) (Hemicellulose Removal (HR), Extractives Removal (ER) and Lignin Removal (LR) %), and the three selected independent variables; concentration, temperature and time (X_1, X_2 and X_3), respectively with the regression coefficients (β_0, β_1, β_2 and β_3) are indicated in Eq. (S1-S4).

$$R\% = \beta_0 + \beta_1 X_1 + \beta_2 X_2 + \beta_3 X_3 + \beta_{12} X_1 \times X_2 + \beta_{13} X_1 \times X_3 + \beta_{23} X_2 \times X_3 \quad \text{(S1)}$$

$$ER\% = -14.4 - 0.207 X_1 + 0.109 X_2 + 7.27 X_3 + 0.00345 X_1 \times X_2 - 0.00171 X_1 \times X_3 - 0.0656 X_2 \times X_3 \quad \text{(S2)}$$

$$HR\% = -8.9 - 0.89 X_1 + 0.276 X_2 + 0.431 X_3 + 0.0266 X_1 \times X_2 - 0.0089 X_1 \times X_3 - 0.00560 X_2 \times X_3 \quad \text{(S3)}$$

$$LR\% = -10.39 - 0.229 X_1 + 0.092 X_2 + 0.221 X_3 + 0.0096 X_1 \times X_2 - 0.00580 X_1 \times X_3 - 0.00190 X_2 \times X_3 \quad \text{(S4)}$$
